# Incidence of *Clostridium difficile* Infection After Sepsis Protocol Antibiotics

**DOI:** 10.5811/westjem.2019.10.42070

**Published:** 2019-10-24

**Authors:** Jordan LaFave, David Levy, Robert Gekle, Robert Bramante

**Affiliations:** Good Samaritan Hospital Medical Center, Department of Emergency Medicine, West Islip, New York

## Abstract

**Introduction:**

The management of sepsis includes the prompt administration of intravenous antibiotics. There is concern that sepsis treatment protocols may be inaccurate in identifying true sepsis and exposing patients to potentially harmful antibiotics, sometimes unnecessarily. This study was designed to investigate those concerns by focusing on in-hospital *Clostridium difficile* infection (CDI), which is a known complication of exposure to antibiotics.

**Methods:**

Our emergency department (ED) recently implemented a protocol to help combat sepsis and increase compliance with the 2017 Sepsis CMS Core Measures (SEP-1) guidelines. In this single-center, retrospective cohort analysis we queried the electronic health record to gather data on nosocomial CDI and antibiotics prescribed over a five-year period to analyze the effect of the introduction of a sepsis protocol order set. The primary goal of this study was to measure the hospital-wide CDI rate for three years prior to implementation of the sepsis bundle, and then compare this to the hospital-wide CDI rate two years post-implementation. As a secondary outcome, we compared the number of antibiotics prescribed in the ED 12 months prior to administration of the sepsis protocol vs 12 months post-initiation.

**Results:**

Over the course of five years, the hospital averaged 9.4 nosocomial CDIs per 10,000 patient hours. Prior to implementation of the sepsis bundle, the average CDI rate was 11.6 (±1.11, 95%) and after implementation the average rate dropped to 6.2 (±1.27, 95%, p<0.01). The mean number of antibiotics ordered per patient visit was 0.33 (±0.015, 95%) prior to bundle activation, and, following sepsis bundle activation, the rate was 0.38 (±0.019, 95%, p<0.01). This accounted for 38% of all ED patient visits receiving antibiotics, a 5% increase after the sepsis bundle was introduced.

**Conclusion:**

In this study, we found that CDI infections declined after implementation of a sepsis bundle. There was, however an increase in the number of patients being exposed to antibiotics after this hospital policy change. There are more risks than just CDI with antibiotic exposure, and these were not measured in this study. Subsequent studies should focus on the ongoing effects of timed, protocolized care and the associated risks.

## INTRODUCTION

The management of sepsis according to the 2017 Surviving Sepsis Campaign guidelines is multifaceted and includes the prompt administration of intravenous antibiotics.^1^ The Centers for Medicare and Medicaid Services (CMS) core measures require administration of antibiotics within three hours of sepsis being identified. It is recommended that broad spectrum antibiotics be used in the initial treatment of sepsis or suspected sepsis based on systemic inflammatory response syndrome (SIRS) criteria.^2^ Although antibiotic treatment may be life-saving, antibiotic exposure has known potential complications, including the risk for developing *Clostridium difficile* infection (CDI).^2^–^4^

CDI has important implications affecting patient mortality, cost, and even potential hospital reimbursement. Studies show mortality of CDI in hospitalized patients ranges from 8–37.2%.^5^ CDI is a major contributor to healthcare expenditure in the United States and was responsible for as much as $4.8 billion U.S. dollars of cost to the health system.^6^ In addition to costs, sepsis performance data are currently being collected by The Joint Commission regarding antibiotic administration in the presentation of SIRS patients, and hospitals may soon find that it will be tied directly to reimbursement.^7^

Compliance with the CMS Sepsis Core Measures (SEP-1), or sepsis bundle, mandates early antibiotic administration. Providers at this facility were encouraged to use an order set that included the SEP-1 required components of sepsis management. Use of antibiotics is known to be associated with the risk of CDI.^2^,^4^ The Infectious Diseases Society of America (IDSA) chose not to endorse the 2016 sepsis guidelines due to concern over excessive antibiotic use and its associated risks, including CDI.^8^ We hypothesized that the incidence of CDI in this hospital would not change after implementation of the sepsis bundle-required antibiotics administration. The primary goal of this study was to measure the hospital-wide CDI rate for three years prior to implementation of the sepsis bundle vs the CDI rate two years post-implementation. As a secondary outcome, we compared the overall number of antibiotics prescribed in the emergency department (ED) 12 months prior to administration of the sepsis protocol vs 12 months post-initiation.

## METHODS

This study was a single-center, retrospective cohort analysis designed to test the hypothesis that the introduction of sepsis bundle antibiotics had no effect on hospital-wide CDI rates. The study was performed in an academic, suburban hospital ED with an annual census of approximately 90,000 visits per year that implemented a protocol on January 15, 2016, to help combat sepsis and increase compliance with SEP-1 guidelines. The facility’s institutional review board approved the study as exempt.

Over the five-year period, the protocol in place to diagnose CDI in the hospital was updated once. Initially, a nosocomial CDI was defined as a positive *C. diff* polymerase chain reaction test. However, in October 2016 the infection control department changed the protocol to a laboratory panel, which includes an enzyme immunoassay test paired with a glutamate dehydrogenase test. If both return positive, the patient was considered to have CDI. If one result was positive and the other negative, the test was considered indeterminate. In that case, a follow-up polymerase chain reaction (PCR) test was sent reflexively to an outside laboratory to evaluate for the presence of two *C. diff*, toxin-related genes (tcdB and tcbC). This follow-up PCR test was considered the final deciding factor for all indeterminate tests.

**Population Health Research Capsule**

What do we already know about this issue?

*There is concern that sepsis treatment protocols may be exposing patients to more antibiotics, and research has shown that antibiotic exposure can be harmful*.

What was the research question?

Does implementation of a sepsis treatment protocol increase hospital-wide incidence of C. difficile infections?

What was the major finding of the study?

*C. difficile infections decreased after implementing a sepsis treatment protocol despite an increase in antibiotic use*.

How does this improve population health?

*Emergency department antibiotic stewardship has long reaching effects in the community. Hospital administrators should consider carefully the effects of the policies they implement*.

We extracted data from the EPIC electronic health record (EHR) with the help of the infection control department, which keeps record of nosocomial hospital infections. The overall number of hospital nosocomial CDI per 10,000 inpatient hours was reviewed and recorded monthly from January 2013–December 2017. For the secondary outcome, we queried the EHR for the daily number of antibiotics ordered on patients ≥18 years of age during their ED stay for the two-year period January 2015–January 2017. The study focused only on antibiotics available to be ordered directly from the sepsis order set (Table 1), and included only those antibiotics ordered by ED providers. Orders placed by inpatient providers were not counted, as the secondary outcome was limited to this protocol’s effect on ED provider antibiotic usage. The sepsis order set went live on January 15 of 2016.

CDI rates three years before January 2016 and two years after were grouped and analyzed for an overall difference in means. For the secondary outcome, we queried, recorded and analyzed the number of antibiotics for one year before and after this date. This period was chosen, as the database for this specific information was limited to one year prior to the time period. Abstractors were blinded to the study’s hypothesis. For analysis, we performed a two-sample t-test assuming equal variances.

## RESULTS

**Figure 1:**
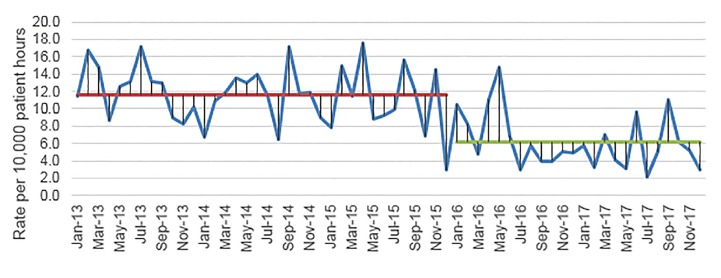
Hospital *Clostridium difficile* rates 2013–2017 before (red) and after (green) sepsis protocol implementation.

**Figure 2:**
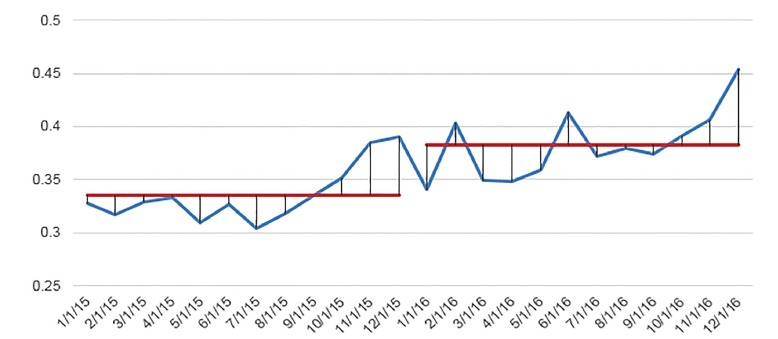
Emergency department antibiotics ordered per patient visit before (1/2015–12/2015) and after (1/2016–12/2016) sepsis protocol implementation.

Over the course of five years, the hospital averaged 9.4 nosocomial CDIs per 10,000 patient hospital hours. Prior to implementation of the sepsis bundle, the average CDI rate was 11.6 (± 1.11, 95%) vs 6.2 (±1.27, 95%) per 10,000 patient hours (Figure 1, Table 2). There was a decrease in the number of hospital-acquired CDIs after the sepsis order set was activated, with a mean monthly decrease of 5.5 nosocomial CDIs per 10,000 patient hours (p<0.01) (Table 2). For the secondary outcome, we measured ED antibiotics ordered the year before and after the sepsis bundle. The mean proportion of patients receiving antibiotics during their ED visit was 0.33 (± 0.015, 95%) prior to bundle activation, with approximately 33% of all patient visits receiving antibiotics. After sepsis bundle implementation, this rose to 0.38 (± 0.019, 95%), or 38% of patient visits receiving antibiotics, for an increase of 5% (p<0.01) (Figure 2, Table 2). Variances were found to be similar across the datasets.

## DISCUSSION

Prior research has shown that antibiotic exposure leads to an increased risk of CDI development.^4^ When earlier CMS recommendations in the management of community-acquired pneumonia outlined strict time periods for antibiotic administration, research on the topic indicated concern that these recommendations could lead to misdiagnosis and inappropriate antibiotic exposure.^9^ CMS has now put a time constraint on management of SIRS-positive patients with presumed or suspected infectious etiology, a protocol that can lead to increased antibiotic administration prior to formal diagnosis and, given the greater antibiotic exposure, a potential increased risk of CDI. As previously noted, the IDSA did not support the 2016 guidelines due to this concern.^8^

This study demonstrated a 5% increase in antibiotic prescriptions for ED patients after sepsis bundle order set initiation. While this supports provider concerns over an increase in antibiotic utilization, hospital CDI rates actually decreased by a mean of 5.5 nosocomial infections per 10,000 patient hours during the study period. Although some practitioners may feel some relief knowing that this study failed to find a CDI epidemic as the result of an overall protocol change, these results may be only one small piece in an overall concerning trend. Instead, it is important to recognize that there are more risks than just CDI with antibiotic exposure, risks that were not measured in this study. Subsequent studies should focus on rate of antibiotic use and the other risks that are involved with these mandated prescribing practices.

There are multiple risk factors for development of CDI other than antibiotic exposure. Some of these include proton-pump inhibitor exposure and poor compliance with the use of personal protective equipment.^10^,^11^ Healthcare facilities frequently implement new practices and staff educational procedures, which may have had an impact on the results and CDI rates.^12^ Although this study showed that rates of antibiotic administration increased there was an unexpected decrease in CDI rates, which may be explained by practice improvements and staff education. While the study was not designed to look at these effects, it provides hope that ongoing facility practices may be mitigating CDI risk despite increased antibiotic exposure.

## LIMITATIONS

Because this was a retrospective analysis limited to a single hospital it comes with the limitations inherit to this type of study. While patients may have been lost to follow-up due to death, utilization of other nearby health systems, or decision to not complete their hospital course, we expect the pre- and post-implementation population to be similarly affected by these confounding factors.

Of note, the protocol for diagnosing nosocomial CDI at this hospital changed during the observation period. A subset analysis of CDI rate before and after implementation of these new diagnostic criteria showed no compelling difference in means in these time periods. As such, this change should have had little impact on our results.

## CONCLUSION

CDI infections decreased after implementation of a sepsis protocol, despite an increased proportion of ED patients receiving antibiotics. There is strong evidence in the literature to support that increased antibiotic exposure leads to an increased rate of CDI. This single-center study did not support that concern. More research is needed to further determine the effects these CMS sepsis bundle implementation guidelines on patient outcomes.

## Figures and Tables

**Table 1:** Antibiotics available in the facility sepsis order set.

Amikacin
Ampicillin-Sulbactam
Azithromycin
Aztreonam
Ceftriaxone
Clindamycin
Gentamicin
Levofloxacin
Meropenem
Metronidazole
Piperacillin-Tazobactam
Vancomycin

**Table 2:** Rate of *Clostridium difficile infection (CDI)* and proportion of patients receiving emergency department antibiotics before and after sepsis bundle implementation.

	Before protocol	After protocol	Change
Mean number hospital-wide CDI per 10,000 patient hours (±SD)	11.6 (±1.11, 95%)	6.2 (±1.27, 95%)	−5.5 (p<0.01)
Mean proportion of patient visits receiving antibiotics (±SD)	0.33 (±0.015, 95%)	0.38 (±0.019, 95%)	+0.05 (p<0.01)
